# Lipid Profile of Activated Macrophages and Contribution of Group V Phospholipase A_2_

**DOI:** 10.3390/biom11010025

**Published:** 2020-12-29

**Authors:** Masaya Koganesawa, Munehiro Yamaguchi, Sachin K. Samuchiwal, Barbara Balestrieri

**Affiliations:** Department of Medicine, Division of Allergy and Clinical Immunology, Brigham and Women’s Hospital, Harvard Medical School, Boston, MA 02115, USA; mkoganesawa@bwh.harvard.edu (M.K.); munehiro1006@yahoo.com (M.Y.); ssamuchiwal@bwh.harvard.edu (S.K.S.)

**Keywords:** Group V phospholipase A_2_, macrophages, lipids

## Abstract

Macrophages activated by Interleukin (IL)-4 (M2) or LPS+ Interferon (IFN)γ (M1) perform specific functions respectively in type 2 inflammation and killing of pathogens. Group V phospholipase A_2_ (Pla2g5) is required for the development and functions of IL-4-activated macrophages and phagocytosis of pathogens. Pla2g5-generated bioactive lipids, including lysophospholipids (LysoPLs), fatty acids (FAs), and eicosanoids, have a role in many diseases. However, little is known about their production by differentially activated macrophages. We performed an unbiased mass-spectrometry analysis of phospholipids (PLs), LysoPLs, FAs, and eicosanoids produced by Wild Type (WT) and *Pla2g5*-null IL-4-activated bone marrow-derived macrophages (IL-4)BM-Macs (M2) and (LPS+IFNγ)BM-Macs (M1). Phosphatidylcholine (PC) was preferentially metabolized in (LPS+IFNγ)BM-Macs and Phosphatidylethanolamine (PE) in (IL-4)BM-Macs, with Pla2g5 contributing mostly to metabolization of selected PE molecules. While Pla2g5 produced palmitic acid (PA) in (LPS+IFNγ)BM-Macs, the absence of Pla2g5 increased myristic acid (MA) in (IL-4)BM-Macs. Among eicosanoids, Prostaglandin E_2_ (PGE_2_) and prostaglandin D_2_ (PGD_2_) were significantly reduced in (IL-4)BM-Macs and (LPS+IFNγ)BM-Macs lacking Pla2g5. Instead, the IL-4-induced increase in 20-carboxy arachidonic acid (20CooH AA) was dependent on Pla2g5, as was the production of 12-hydroxy-heptadecatrienoic acid (12-HHTrE) in (LPS+IFNγ)BM-Macs. Thus, Pla2g5 contributes to PE metabolization, PGE_2_ and PGD_2_ production independently of the type of activation, while in (IL-4)BM-Macs, Pla2g5 regulates selective lipid pathways and likely novel functions.

## 1. Introduction

Macrophages are heterogeneous cells that contribute to the pathogenesis of infectious disease, type 2 immune responses, and metabolic disorders [1,2,3]. Macrophages exposed to pathogen-associated molecular patterns (PAMPS), endogenously formed danger-associated molecular patterns (DAMPs), and to the microenvironmental milieu rearrange their repertoire of cytokines and chemokines and contribute to the immune responses [4,5]. In vitro, the immune responses of macrophages are simplified by LPS+Interferon (IFN)γ (M1) and Interleukin (IL)-4 (M2) polarization [6,7]. Although it is unlikely that macrophages in vivo exist in mutually exclusive phenotypes [2,4], the polarization paradigm has helped to dissect critical immune features of macrophages. M2 macrophages develop during type 2 inflammation, prompted by Type 2 cytokines including IL-4 and IL-13, and they are characterized by the production of CCL22, CCL17, Arginase-1, and Transglutaminase 2 (TGM2) [7,8,9]. M1 macrophages develop during infection and are equipped with an array of cytokines, including IL-12, TNFα, and reactive oxygen species (ROS), to fight pathogens. Metabolically, macrophage polarization is distinguished by activation of aerobic glycolysis in M1 macrophages, particularly when M1 activation is achieved by IFNγ, in addition to LPS [10] and fatty acid oxidation in M2 [11]. Fatty acids (FAs) are involved in the remodeling of membrane phospholipid (mPLs), macrophage development and polarization [12,13,14], and they are essential for phagocytosis by fully differentiated macrophages [14]. Furthermore, polyunsaturated fatty acids (PUFAs) are the precursors of eicosanoids, pro- or anti-inflammatory lipid mediators, which are generated through three main pathways, cyclooxygenase (COX), lipoxygenase (LOX), and monooxygenases like CYP450 (CYP). Even before the identification of macrophage polarization and their spectrum of phenotypes, stimuli like Granulocyte macrophage colony-stimulating factor (GM-CSF) and IL-4 reportedly induced 5-lipoxygenase (5-LOX) activation in macrophages [15,16], the limiting step in the generation of Cysteinyl leukotrienes (CysLTs), major mediators of inflammation [17]. COX-derived Prostaglandin E_2_ (PGE_2_) and PGD_2_ may have pro- or anti-inflammatory functions associated with LPS or IL-4 immune responses [18,19,20]. CYP2J2-derived epoxyeicosatrienoic acids (EETs) have opposite roles in human monocyte-macrophages depending on the activation state of the macrophages [21], while CYP omega-oxidase 20-hydroxy-eicosatetraenoic acid (20-HETE) and its metabolite 20-carboxy arachidonic acid (20CooH AA) may have anti-inflammatory properties due to the ability to activate Peroxisome Proliferator-Activated Receptor α (PPARα) and PPARγ [22] while also activating G Protein-Coupled Receptor 75 (GPR75) [23]. However, whether M1 or M2 activated macrophages differentially produce FAs and eicosanoids has not been fully elucidated, but is potentially critical to understand how lipids produced by macrophages may contribute alone or in combination to the many diseases involving activated macrophages.

The release of FAs and eicosanoids requires the activity of at least one of the phospholipases A_2_ (PLA_2_s) enzymes. PLA_2_s hydrolyze membrane phospholipids to generate FAs, including saturated fatty acids, monounsaturated fatty acids (MUFAs), and polyunsaturated fatty acids (PUFAs) and their metabolites eicosanoids, and lysophospholipids (LysoPLs). As PLA_2_s have substrate specificities and cell-expression preferences, likely, activated macrophages would preferentially express one or more PLA_2_s and, thus, contribute to the generation of selective bioactive lipids. It is worth noting that the constitutive and ubiquitous group IVA cytosolic PLA_2_ (cPLA_2_α or Pla2g4a), which preferentially releases Arachidonic Acid (AA), is expressed more abundantly in M1 macrophages than in M2 macrophages [24]. Of the secretory PLA_2_ family members, group V PLA_2_ (Pla2g5) is expressed in macrophages [25] and other cells [26]. Several studies, including ours, have reported the presence of Pla2g5 mRNA and protein in bone marrow-derived macrophages (BM-Macs), peritoneal macrophages, human monocyte-derived macrophages, macrophage-cell lines [27]. Pla2g5 can potentiate Pla2g4a activation and AA release in macrophages activated with Toll-Like Receptor (TLR) agonists [28] and is required for phagocytosis and killing of pathogens [27,29]. In vivo, its absence results in increased LPS inflammation and mortality following Candida infection [30,31]. On the other hand, Pla2g5 is induced by IL-4 in mouse and human macrophages [20,32,33,34] and is expressed together with TGM2 in macrophages present in human nasal polyps of subjects with chronic rhinosinusitis [20]. Furthermore, Pla2g5 contributes to M2 macrophage development and functions [20,33,34]. Indeed, in allergic type 2 inflammation, Pla2g5 potentiates inflammation and activation of target cells, including T-cells and innate lymphoid cells type 2 (ILC2s) through the generation of the linoleic acid (LA) and oleic acid (OA) [32,34]. However, the contribution of Pla2g5 to lipid generated during M1 and M2 polarization is still not known, but is potentially critical to account for the functions of M1 and M2 macrophages.

To investigate the lipid generated during macrophage polarization, we activated BM-Macs with IL-4 or LPS+IFNγ and analyzed FAs, PLs, LysoPLs, and eicosanoids by gas chromatography coupled to mass spectrometry (GC-MS) and liquid chromatography coupled to mass spectrometry (LC-MS). We found that macrophages activated by LPS+IFNγ or IL-4 rearrange their membrane phospholipid composition by decreasing phospholipid content, particularly Phosphatidylethanolamines (PE) during IL-4 activation and Phosphatidylcholine (PC) during LPS+IFNγ activation. Furthermore, in M2 macrophages, the absence of Pla2g5 increased PE 34:2, PE 36:2, PE 38:4, and PE 38:5, while in M1 macrophages, there was a significant increase in PC 34:1 and PC 36:2. LysoPLs were not significantly modified during macrophage activation in BM-Macs lacking Pla2g5. We also found that Pla2g5 regulated the release of selective saturated fatty acids in activated BM-Macs. Furthermore, PGD_2_ and PGE_2_ were significantly induced by Pla2g5 in both M1 and M2 activation, while 20CooH AA production was dependent on Pla2g5 only in M2. Therefore, it is likely that the combined action of the lipids produced by M1 and M2 macrophages may contribute to the functions of macrophages in different settings. Identifying potential lipid signatures in M1 and M2 macrophages could uncover new pathways critical for the development, persistence, or reduction of inflammation. Some of these functions may rely on the presence or absence of Pla2g5 in macrophages.

## 2. Materials and Methods

### 2.1. Macrophage Cultures

BM-Macs were generated as previously described [34]. Briefly, BM cells were collected from femurs and tibiae of mice of Wild Type (WT) and *Pla2g5*-null mice [35]. The disaggregated cells were counted and suspended in complete medium (Dulbecco’s Modified Eagle Medium (DMEM) F12, 5% Fetal Bovine Serum (FBS), 100 U mL^−1^ penicillin, 100 mg mL^−1^ streptomycin, 0.1 mM nonessential amino acids, 2 mM L-glutamine and 0.05 mM 2-mercaptoethanol (2-ME)) at a concentration of 4.0 × 10^6^ cells per mL in 10 mL in 100 mm Petri dish. WT and *Pla2g5*-null BM-macrophages were cultured for 7 days in 50 ng mL^−1^ murine recombinant-Macrophage Stimulating Factor (rM-CSF) (PeproTech). Macrophages were activated with IL-4 or LPS+IFNγ (PeproTech) for 24 h, as previously described [32]. Supernatants and adherent cells were collected [20,34], frozen, and shipped for analysis by mass spectrometry.

Human monocyte-derived macrophages were generated as previously described [20]. Briefly, leukocyte-enriched buffy coat from healthy donors was overlaid on Ficoll-Paque Plus (GE Healthcare, Buckinghamshire, UK) and centrifuged at 600× *g* for 20 min. The mononuclear layer at the interface was collected, washed, and counted. Monocytes were isolated by negative selection (Miltenyi Biotec, Auburn, CA, USA) and plated at 2.2 × 10^5^ cells/cm^2^ in 100 mm Petri dishes. Monocytes were cultured for 13 d in complete medium (RPMI 1640, 10% FBS, 2 mM L-glutamine, 100 U/mL penicillin, 100 mg/mL streptavidin, 10% nonessential amino acids, 1% 4-(2-hydroxyethyl)-1-piperazineethanesulfonic acid (HEPES), 1% sodium pyruvate, and 50 mM 2-ME supplemented with 50 ng mL^−1^ human rGM-CSF (R&D Systems, Minneapolis, MN, USA) [36]. To knock down PLA2G5, monocytes were cultured for 13 d in rGM-CSF, then they were transfected with human PLA2G5 ON-TARGET Plus SMART Pool siRNA or nontargeting vector ONTARGET Plus Control Pool (1000 nM; GE Dharmacon, Lafayette, CO, USA) as previously described [20]. To activate macrophages, cells were polarized for 24 h in complete medium, supplemented with 40 ng mL^−1^ human IL-4 (R&D Systems). Cell-free supernatants were collected, frozen and shipped for analysis by mass spectrometry.

### 2.2. Mass Spectrometry of Lipids

Lipid analysis was performed at the University of California San Diego Lipidomics Core [37]. For analysis of eicosanoids samples, the amount of sample used was 200 μL, and 100 μL internal standard was added. Lipids were extracted with Solid Phase Extraction (SPE): Strata-x polymeric reverse phase columns (8B-S100-UBJ; Phenomenex, Torrance, CA, USA). The following was added to each column: 100% MeOH, 100% H_2_O, sample, 10% MeOH, and 100% MeOH for elution. Samples were dried with a Speed-Vac (Thermo Scientific) and taken up in 100 mL buffer A (63% H_2_O, 37% acetonitrile, 0.02% acetic acid). Five microliters were injected into the ultra-high performance liquid chromatography system. The analysis was performed with a mass spectrometer (6500 Qtrap; Sciex, Framingham, MA, USA) [38]. For analysis of phospholipids, samples were extracted via Bleigh Dyer. Samples were dried down and taken up in 50 µL buffer A (59/40/1 isopropanol (IPA)/hexane (HEX)/H_2_0 with 10 mM NH_4_OAC). Then, 10 µL were injected into UPLC-MS/MS. The first number of the phospholipids designates the total number of carbons and the second number after the colon indicates the total number of double bonds. For example, PC 36:4 indicates a phospholipid molecule with a total of 36 carbons and 4 double bonds. Lysophosphatidylcholine (LysoPC)-O and PC-O indicate ether-linked fatty acids in the sn-1 position. We measure isobaric species. For example, PC 36:4 can consist of either PC (18:2; 18:2) or PC (16:0; 20:4). Similarly, we did not discriminate between 34:0 PC and 36:7 PC-O—both have identical molecular masses and are not separated by LC-MS.

FA analysis was performed according to a previously published method [39]. Briefly, the cell pellet was homogenized in 500 mL of PBS/10% methanol. An aliquot of 200 mL corresponding to about 0.5 × 10^6^ cells was withdrawn and a cocktail of internal standards consisting of 15 deuterated fatty acids was added. The extraction was initiated with 500 mL of methanol and 25 mL of 1 N HCl and a bi-phasic solution is formed by addition of 1.5 mL of isooctane. The phases are separated by centrifugation and the isooctane phase containing the FFAs fraction was removed. The extraction is repeated once and the combined extracts were evaporated to dryness. The FFAs were derivatized with pentafluorobenzyl (PFB) bromide and the resulting fatty acid PFB esters were analyzed by gas chromatography/mass spectrometry using a negative chemical ionization mode (Agilent 6890N gas chromatograph equipped with an Agilent 5973 mass selective detector; Agilent, Santa Clara, CA, USA). Standard curves for each of the fatty acids were acquired in parallel using identical conditions. The quantitative assessment of fatty acids in a sample was achieved by comparison of the mass spectrometric ion signal of the target molecule normalized to the internal standard with the matching standard curve according to the isotope dilution method and by protein content.

### 2.3. qPCR

Total RNA was isolated from lysate with the RNeasy Micro Kit (Qiagen, Louisville, KY, USA), reverse transcribed into cDNA (High-Capacity cDNA Reverse Transcription Kit; Thermo ARTICLES science-Applied Biosystems, Foster City, CA, USA) and measured by real-time PCR with the use of SYBR^®^ Green/ROX master mix (SABiosciences, Frederick, MD, USA) on an Mx3005P thermal cycler (Stratagene, Santa Clara, CA, USA). The ratio of each mRNA relative to the Glyceraldehyde-3-Phosphate Dehydrogenase (GAPDH) mRNA was calculated with the ΔΔCt threshold cycle method. The mouse primers used were GAPDH F: 5′-TCAACAGCAACTCCCACTCTTCCA-3′; R: 5′-ACCCTGTTGCTGTAGCCGTATTCA-30′ Pla2g5 F: 5′-TGGTTCCTGGCTTGCAGTGTG-3′; R: 5′-TTCGCAGATGACTAGGCCATT-3′ [34]. Real-time PCR products were run on a 1.5% agarose gel and visualized using chemilmager 4400 fluorescence system (Alpha Innotech, Missouri, TX, USA).

### 2.4. Statistical Analysis

Comparisons between two groups were made by using an unpaired Student’s *t* test, and other comparisons were made with two-way ANOVA with Tukey, Sidak, or Dunnett correction for multiple comparisons. Comparisons were performed with Prism software (GraphPad, San Diego, CA, USA). Data are expressed as mean ± standard error of the mean (SEM); significance was set at *p* < 0.05.

## 3. Results

### 3.1. Phospholipid Metabolism in Bone Marrow-Derived Macrophages Activated by IL-4 or LPS+IFNγ

**Figure 1:**
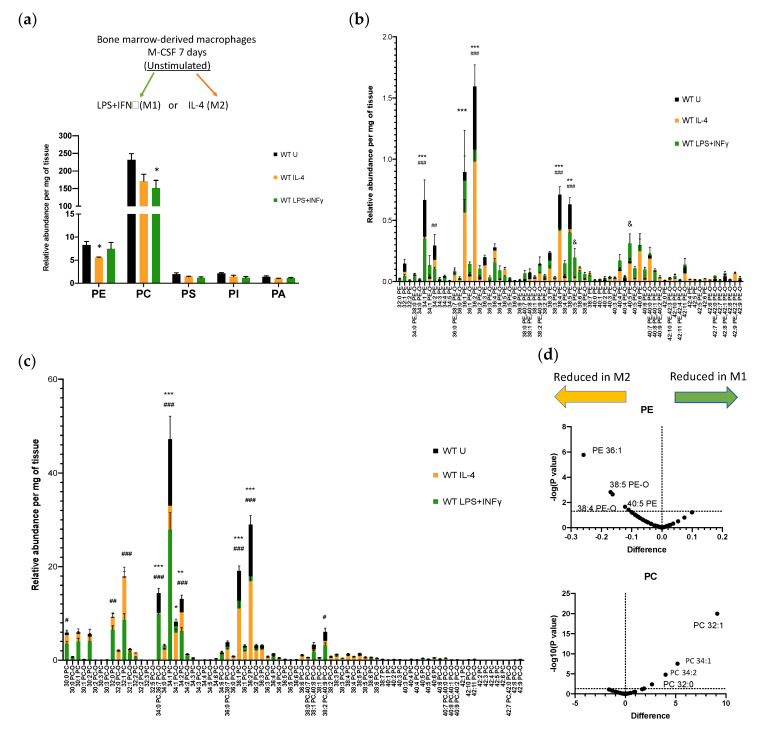
Phospholipid metabolism in bone marrow-derived macrophages (BM-Macs). Wild Type (WT) BM-Macs were Unstimulated (U) or stimulated with Interleukin (IL)-4 or LPS+ Interferon (IFN)γ for 24 h, supernatants were removed and collected for analysis by liquid chromatography coupled to mass spectrometry (LC-MS). (**a**) * *p* < 0.05 of (U) vs. IL-4 or LPS+IFNγ BM-Macs. (**b**,**d**) Phosphatidylethanolamine (PE) molecules or (**c**,**d**) phosphatidylcholine (PC) molecules are identified by the binding of fatty acids which are described by the numbers of carbons and double bonds. PE-O and PC-O indicate ether linked fatty acids in the sn-1 position. The data are shown volcano plot of mean (**d**) or graph (**a**–**c**) of mean and standard error of three independent determinations. *** *p* < 0.0001, ** *p* < 0.005 (IL-4)BM-Macs reduction vs. (U)BM-Macs; ^###^ *p* < 0.0001, ^##^ *p* < 0.005, ^#^ *p* < 0.05 (LPS+IFNγ)BM-Macs reduction vs. (U)BM-Macs; ^&^ *p* < 0.05 increase in (LPS+IFNγ)BM-Macs vs. (U)BM-Macs. *p*-values were obtained by two-way ANOVA with Dunnett’s correction for multiple comparisons.

To investigate the production of bioactive lipids in activated BM-Macs, we first asked whether, during macrophage activation, there were changes in the composition of membrane phospholipids (PLs), substrates of PLA_2_. We cultured mouse BM-Macs for 7 days in rM-CSF and 24 h with IL-4 or LPS+IFNγ, hereafter referred to as (IL-4)BM-Macs or (LPS+IFNγ)BM-Macs, respectively, for M2 or M1 (Figure 1a, inset), to distinguish them from other in vitro derived M2 or M1 macrophages [40]. The supernatants were collected and the phospholipid species analyzed by mass spectrometry. Five PL species were detected in resting WT BM-Macs. Phosphatidylcholine (PC) was the most abundant, followed by phosphatidylethanolamines (PE), while phosphatidylinositol (PI), phosphatidylserine (PS), and phosphatidic acid (PA) contributed minimally to membrane composition (Figure 1a, black columns). Compared to WT unstimulated (U)BM-Macs, PE was significantly decreased in WT (IL-4)BM-Macs, while PC was significantly reduced in (LPS+IFNγ)BM-Macs (Figure 1a). PC, PS, PI, and PA did not change following activation. PG species were undetectable. These data suggest that PE could be the prevalent substrate for a PLA_2_ in (IL-4)BM-Macs and PC in (LPS+IFNγ)BM-Macs, although other phospholipases could contribute to remodeling of membrane phospholipids.

Next, we wanted to ascertain whether, among PE or PC species, there were preferred phospholipid molecules decreased during each of the macrophage activation models and, therefore, preferred bioactive lipid produced. We analyzed 69 PE molecules. In WT (U)BM-Macs, PE 36:2 was the most abundant. Compared to WT (U)BM-Macs, PE 34:1, PE 36:2, PE 38:4, and PE 38:5 were significantly decreased in both (IL-4) and (LPS+IFNγ)BM-Macs, and PE 34:2 was significantly reduced in (LPS+IFNγ)BM-Macs while PE 36:1 was significantly reduced in (IL-4)BM-Macs (Figure 1b). These data indicate that long-chain PUFAs, including AA, are liberated from PE in both IL-4 and LPS+IFNγ activation. Instead, PE-O 38:5 and PE 40:5 were significantly increased in WT (LPS+IFNγ)BM-Macs compared to WT (U)BM-Macs, likely indicating re-acylation. Of the 80 PC species analyzed (Figure 1c), PC 34:1 was the most abundant in WT (U)BM-Macs and was reduced in both (IL-4) and (LPS+IFNγ)BM-Macs as were PC 34:2, PC 36:1, PC 36:2 PC-O 34:0/PC-O 36:7. LPS+IFNγ activation resulted in the selective reduction of PC 30:0, PC 32:0, PC 32:1, and PC 38:2/PC-O 40:9 compared to (U)BM-Macs. A direct comparison of PE or PC molecules in (IL-4)BM-Macs versus (LPS+IFNγ)BM-Macs confirmed that PE species were preferentially reduced and likely metabolized in (IL-4)BM-Macs and PC species in (LPS+IFNγ)BM-Macs (Figure 1d). These results suggest that one or more PLA_2_ may hydrolyze preferred substrates in IL-4 or LPS+IFNγ activated macrophages.

**Figure 2:**
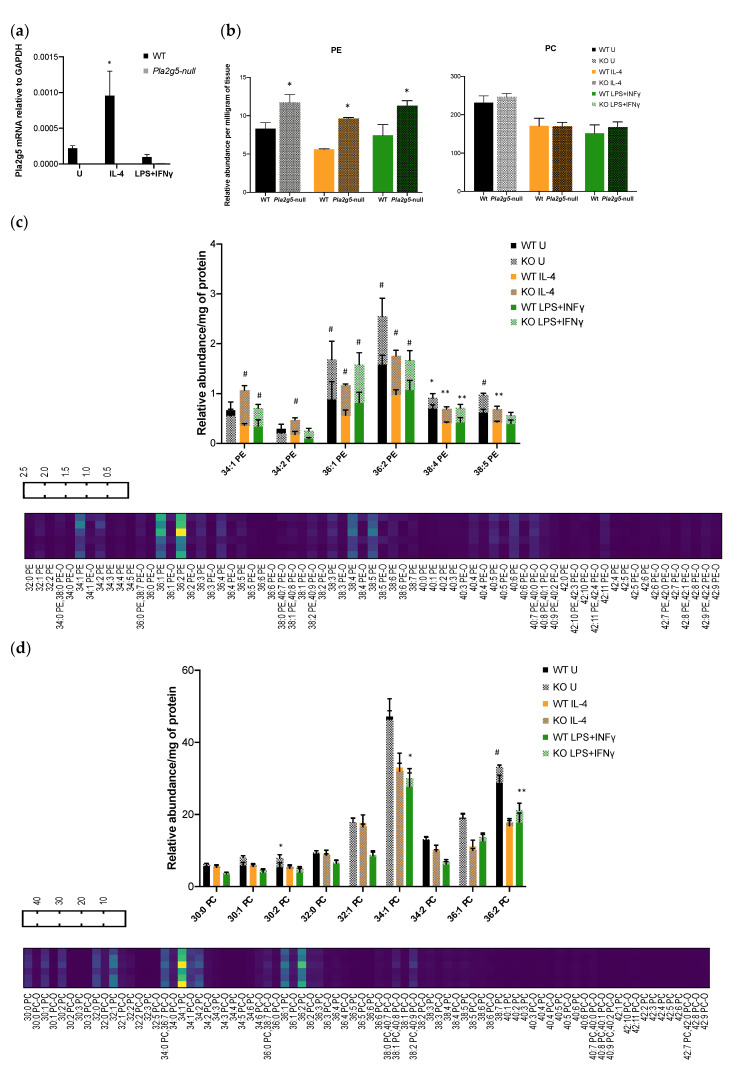
PE and PC molecules from WT and *Group V phospholipase A_2_ (Pla2g5)*-null BM-Macs determined by LC-MS. Expression of Pla2g5 mRNA relative to Glyceraldehyde-3-Phosphate Dehydrogenase (GAPDH) measured by qPCR in WT (black bars) or *Pla2g5*-null BM-Macs (grey bars), Unstimulated (U) or stimulated with IL-4 or LPS+IFNγ (**a**). BM-Macs isolated from WT (solid bars) or *Pla2g5*-null (dotted bars) mice were Unstimulated (U) or treated with IL-4 or LPS+IFNγ for 24 h and (**b**) total PC and PE, (**c**) PE, or (**d**) PC molecules are reported. The data are shown as a heatmap of mean or graph of mean and standard error of three independent determinations. ^#^ *p* < 0.0001, ** *p* < 0.005, * *p* < 0.05 by two-way ANOVA with Sidak’s or Tukey’s correction for multiple comparisons.

Since Pla2g5 is induced by IL-4 [32,34] and prefers PE as a substrate at least in human monocyte-macrophages activated by IL-4 [33], we wanted to understand whether the changes in membrane phospholipid were due at least partially to Pla2g5, particularly in IL-4 BM-Macs. We confirmed that Pla2g5 is induced in mouse BM-Macs by IL-4, but not LPS+IFNγ (Figure 2a) as previously shown [32]. In *Pla2g5*-null BM-Macs, PE was significantly increased in unstimulated, IL-4 and LPS+IFNγ stimulated BM-Macs compared to equally treated WT BM-Macs (Figure 2b) while PC was similar in both WT and *Pla2g5*-null BM-Macs independently of the activation state. Furthermore, compared to equally treated WT BM-Macs, the percentage increase of PE was 71.7 ± 21.1% in (IL-4)BM-Macs, 41.1 ± 2.4% in (U)BM-Macs and 59.7 ± 26.1% in (LPS+IFNγ)BM-Macs (data not shown). These data suggests that PE is the preferred substrate of Pla2g5 in (IL-4)BM-Macs and that the low expression level of Pla2g5 in (U)BM-Macs and (LPS+IFNγ)BM-Macs is sufficient to mobilize PE.

To understand whether Pla2g5 was active on selective PE or PC molecules, we analyzed PE and PC species in *Pla2g5*-null BM-Macs compared to WT BM-Macs. PE 36:1 (18:0, 18:1), PE 36:2 (18:0, 18:2), and PE 38:4 (18:0, 20:4) were increased in BM-Macs lacking Pla2g5 independently of the activation state. PE 34:1(16:0; 18:1), PE 34:2 (16:0,18:2), and PE 38:5 (16:0, 22:5; 18:1, and 20:4) were significantly increased in *Pla2g5*-null (IL-4)BM-Macs while PE 34:1 (16:0; 18:1) was increased in *Pla2g5*-null (LPS+IFNγ)BM-Macs, compared to their respective controls (Figure 2c). In the PC group, PC 30:2 and PC 36:2 were increased in *Pla2g5*-null (U)BM-Macs compared to WT (U)BM-Macs (Figure 2d), while PC 34:1 and PC 36:2 were increased in *Pla2g5*-null (LPS+IFNγ)BM-Macs compared to relative controls. These data suggest that Pla2g5 preferentially metabolized PE molecules in both (IL-4)BM-Macs and (LPS+IFNγ)BM-Macs and that its activity on PC is restricted to (LPS+IFNγ)BM-Macs.

### 3.2. Lysophospholipid and Fatty Acid Generation in Activated BM-Macs

To further investigate the changes in bioactive lipids during macrophage activation, we analyzed LysoPLs by LC-MS. A heat map of LysoPLs generated in BM-Macs showed that compared to unstimulated WT BM-Macs, LysoPC and Lysophosphatidylethanolamine (LysoPE) are substantially reduced following activation with either IL-4 or LPS+IFNγ (Supplemental Figure S1). This is likely due to LysoPL being rapidly metabolized or re-acylated into membrane phospholipids [41]. Furthermore, there was no significant difference between WT and *Pla2g5*-null BM-Macs in either LysoPE or LysoPC molecules in any stimulation (Supplemental Figure S1).

**Figure 3:**
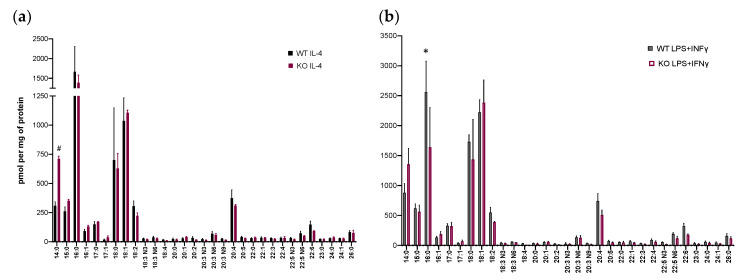
FAs release by activated WT and *Pla2g5*-null BM-Macs. BM-Macs isolated from WT or *Pla2g5*-null mice were Unstimulated (U) or treated with IL-4 (**a**) or LPS+IFNγ (**b**) for 24 h. The production of FAs measured by gas chromatography coupled to mass spectrometry (GC-MS) in WT and *Pla2g5*-null BM-Macrophages. Data are from three independent experiments. Two Way ANOVA followed by Fisher’s LSD post-test. ^#^ *p* < 0.005, * *p* < 0.01.

FAs are bioactive lipids and the second product of PLA_2_ activity on membrane phospholipids. We analyzed 33 FAs by GC-MS [42]. WT BM-Macs showed that FAs generated in (LPS+IFNγ)BM-Macs and (IL-4)BM-Macs were reduced compared to (U)BM-Macs (data not shown), likely because they are being metabolized to their final products, eicosanoids. However, to understand whether Pla2g5 differentially contributes to FAs generated by activated macrophages, we compared FAs produced by WT BM-Macs and *Pla2g5*-null BM-Macs activated by IL-4 or LPS+IFNγ (Figure 3a,b). There was a trend toward reduction in LA (18:2) in *Pla2g5*-null (IL-4)BM-Macs compared to WT (IL-4)BM-Macs (Figure 3a). However, myristic acid (MA; 14:0) was significantly higher in *Pla2g5*-null (IL-4)BM-Macs compared to WT (IL-4)BM-Macs. Instead, compared to WT (LPS+IFNγ)BM-Macs, *Pla2g5*-null (LPS+IFNγ)BM-Macs had a significant reduction palmitic acid (PA; 16:0), and trends toward a reduction in AA (20:4) (Figure 3b).

### 3.3. Differential Eicosanoid Generation in Bone Marrow-Derived Macrophages Activated by IL-4 or LPS+IFNγ

**Figure 4:**
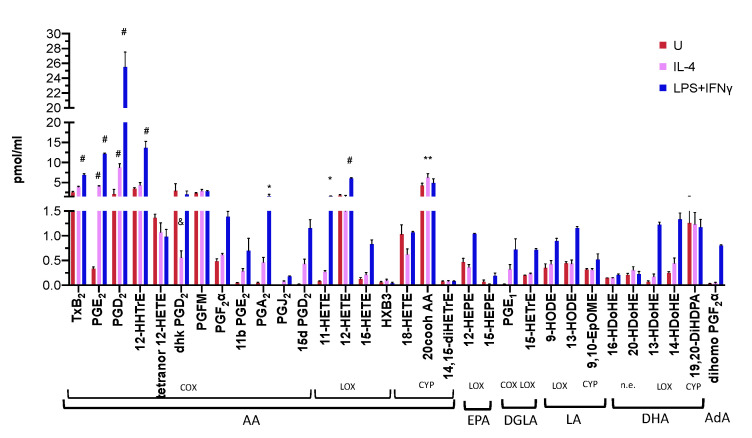
Eicosanoids produced by activated WT BM-Macs. Analysis by LC-MS/MS of cell-free supernatants obtained from WT BM-Macs unstimulated (U) and stimulated for 24 h with IL-4 or LPS+IFNγ. Shown is mean and standard error of eicosanoids produced by WT BM-Macs unstimulated (U, Purple bars) or activated with IL-4 (Red bars) or LPS+IFNγ (Blue bars). Eicosanoids were identified based on matching chromatographic retention times (RTs), fragmentation patterns, and six characteristic and diagnostic ions. Cyclooxygenase (Cox), Lipoxygenase (LOX) Cytochrome P450 (CYP), non-enzymatically (n.e.), Thromboxane B_2_ (TxB_2_), Prostaglandin E_2_ (PGE_2_), prostaglandin D_2_ (PGD_2_), 13,14-dihydro-15-keto prostaglandin D_2_ (dhk PGD_2_), 20-Carboxy-arachidonic acid (20CooH AA), 12-Hydroxyheptadecatrenoic acid (12-HHTrE), Prostaglandin F metabolite (PGFM), Hepoxilin B3 (HXB3), HODE (Hydroxy-octadecadienoic acid), EpHOME (epoxy-octadecenoic acid), HETrE (hydroxy-eicosatrienoic acid), HDoHE (hydroxy-docosahexaenoic acid), DiHDPA (dihydroxydocosa-pentaenoic acid), DiHETrE (dihydroxy-eicosatrienoic acid), HEPE (Hydroxy-eicosapentaenoic acid), HETE (Hydroxy-eicosatetraenoic acid), HPETE (Hydroperoxy-eicosatetraenoic acid), arachidonic acid (AA), LA (linoleic acid), DGLA (Dihomo-gamma linolenic acid) EPA (eicosapentaenoic acid), DHA (docosahexaenoic acid), AdA (Adrenic acid). ^#^ *p* < 0.0001, ** *p* < 0.001 * *p* < 0.05 by two-way ANOVA with Dunnett’s correction for multiple comparisons of three independent determinations.

Because LysoPLs and FAs were reduced in activated BM-Macs compared to unstimulated BM-Macs, we reasoned that eicosanoid could be increased. To verify the effects of IL-4 and LPS+IFNγ on the eicosanoids generated in BM-Macs, we performed an unbiased lipid profile by LC-MS/MS. We detected 32 of the 154 analyzed lipids. The eicosanoids produced by WT BM-Macs unstimulated and following IL-4 and LPS+IFNγ activation originated from AA (C20:4), Eicosapentaenoic acid (EPA, 20:5), Dihomo-γ linolenic acid (DGLA, 20:3), linoleic acid (LA, 18:2), Docosahexaenoic acid (DHA, 22:6), and Adrenic acid (AdA, 22:4) (Figure 4). WT unstimulated BM-Macs produced AA-derived metabolites generated through the three major enzymatic pathways COX, LOX, CYP, and non-enzymatically (n.e.). PGD_2_, Thromboxane B_2_ (TxB_2_), 12-Hydroxy-eicosatetraenoic acid (12-HETE), 20CooH AA, 12-hydroxy-heptadecatrienoic acid (12-HHTrE), 13,14-dihydro-15-keto prostaglandin D_2_ (dhk PGD_2_), and Prostaglandin F metabolite (PGFM) were the most abundant (Figure 4). Compared to WT (U)BM-Macs, both (IL-4) and (LPS+IFNγ)BM-Macs had a significant increase in the production of the COX metabolites PGE_2_ and PGD_2_. Instead, compared to WT (U)BM-Macs, in (LPS+IFNγ)BM-Macs, there were increased amounts of TxB_2_, PGA_2_, 12-HHTrE (COX metabolites), 11-HETE (non-enzymatic product), and 12-HETE (LOX product). Furthermore, in (LPS+IFNγ)BM-Macs, there was a trend in increasing 13-hydroxy-docosahexaenoic acid (13-HDoHE), 14-HDoHE, and dihomo-PGF_2_α. Also, 20CooH AA, a metabolic product of CYP-generated 20-HETE, was significantly increased in (IL-4)BM-Macs compared to (U)BM-Macs. These data suggest that while LPS+IFNγ robustly activates COX and LOX pathways, IL-4 activation of BM-Macs produces selective COX metabolites while also sustaining CYP metabolism as indicated by the increase in 20CooH AA.

### 3.4. Pla2g5 Contributes to Selective Eicosanoid Generation in Unstimulated And Activated Macrophages

**Figure 5:**
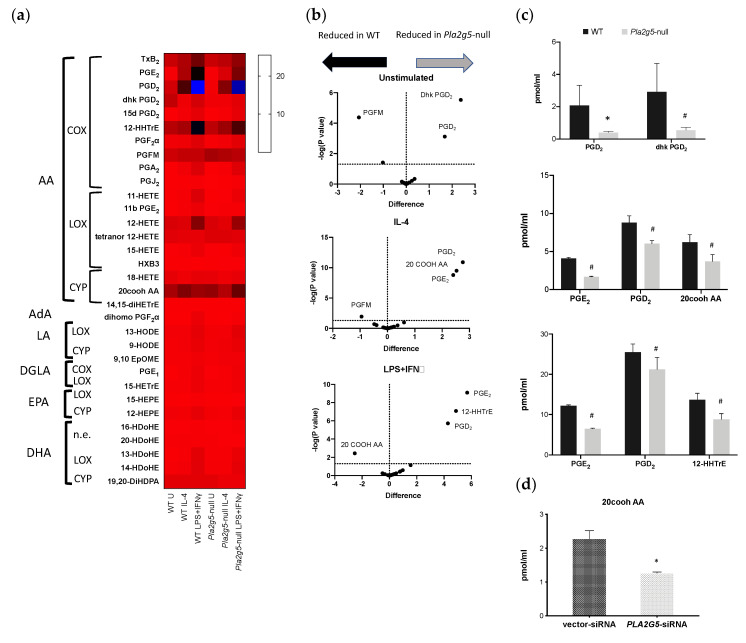
Contribution of Pla2g5 to eicosanoids produced by activated Macs. Analysis by LC-MS/MS of cell-free supernatants obtained from (**a**–**c**) WT and *Pla2g5*-null BM-Macs unstimulated (U) and stimulated for 24 h with IL-4 or LPS+IFNγ or (**d**) 20CooH AA measured in the supernatants collected from vector- and *PLA2G5*-siRNA transfected human monocyte-derived macrophages (h-Macs) stimulated with IL-4. Shown are (**a**) heatmap (**b**) volcano plots of mean values (**c**,**d**) graphs of mean and standard error of eicosanoids produced. ^#^ *p* < 0.0001, * *p* < 0.05 by two-way ANOVA with Sidak’s (**c**) correction for multiple comparisons or *t*-Test (**d**) of three independent determinations.

To better understand the contribution of Pla2g5 to eicosanoid generation during macrophage polarization, we compared eicosanoid generation in WT BM-Macs and *Pla2g5*-null (U)BM-Macs and (IL-4) or (LPS+IFNγ)BM-Macs (Figure 5). Compared to WT (U)BM-Macs, in *Pla2g5*-null (U)BM-Macs there was a reduction in PGD_2_, and dhk PGD_2_ (Figure 5b,c upper panels). Furthermore, compared to WT (IL-4)BM-Macs, in *Pla2g5*-null (IL-4)BM-Macs, there was a significant reduction of PGE_2_, PGD_2_, and 20CooH AA (Figure 5b,c middle panels), the three metabolites that were significantly induced by IL-4 in WT BM-Macs. Stimulation of macrophages with LPS+IFNγ revealed that *Pla2g5*-null (LPS+IFNγ)BM-Macs have a significant reduction in PGE_2_, PGD_2_, and 12-HHTrE, compared to WT (LPS+IFNγ)BM-Macs (Figure 5b,c lower panels). Unexpectedly, the absence of Pla2g5 in BM-Macs resulted in an increase of selected lipids: in unstimulated and in IL-4 activated BM-Macs lacking Pla2g5, PGFM was increased compared to equally treated WT BM-Macs (Figure 5b), while in (LPS+IFNγ)BM-Macs lacking Pla2g5, 20CooH AA was increased compared to equally stimulated WT BM-Macs, likely as a result of reduced metabolism through the COX and LOX pathways. These data suggest that Pla2g5 is involved in the generation of selected metabolites of the COX pathway (IL-4)BM-Macs and metabolites of the COX and LOX pathways in (LPS+IFNγ)BM-Macs, while CYP-induced production of 20CooH AA seemed to be associated with Pla2g5 function in IL-4 activated macrophages. Cysteinyl-leukotrienes (Cys-LTs) were not detected in any conditions in either WT or *Pla2g5*-null BM-Macs.

To ascertain the contribution of human group V PLA_2_ (*PLA2G5*) to eicosanoid generation in human monocyte-derived macrophages (h-Macs) activated by IL-4, we analyzed the lipidomic data set previously generated that demonstrated reduced PGE_2_ generation in the absence of *PLA2G5* [20]. When compared with vector-treated (IL-4)h-Macs, *PLA2G5*-siRNA-treated (IL-4)h-Macs showed significant reduction of 20CooH AA (*p* = 0.014) (Figure 5d). Thus, Pla2g5 supports 20CooH AA production in mouse BM-Macs and h-Macs activated by IL-4.

## 4. Discussion

**Figure 6:**
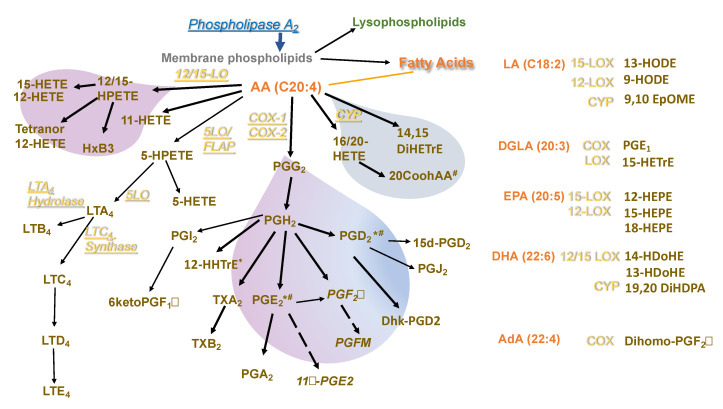
Flow-chart of bioactive lipids produced in activated BM-Macs and contribution of Pla2g5. Phospholipase A_2_ hydrolysis of membrane glycerophospholipids generates lysophospholipids, and free fatty acids including polyunsaturated fatty acids (PUFAs) which are then metabolized to eicosanoids through 3 enzymatic pathways: COX, LOX, and monooxygenases like CYP. Depicted in yellow are the enzymes; in brown the metabolites; and in orange are the fatty acids. The purple bubble depicts eicosanoids generate mainly through activation by LPS+IFNγ in blue those generated mainly through IL-4 activation, and in purple–blue are those in common. Metabolites reduced in *Pla2g5*-null (LPS+IFNγ)BM-Macs (*) or (IL-4)BM-Macs (^#^). FLAP (5-lipoxygenase-activating protein).

The study of macrophage activation has received increasing attention because of its potential implications in the development and treatment of multiple diseases. Macrophage activation is exemplified by the polarization of macrophages with IL-4 or LPS+IFNγ, each leading to differential gene expression, metabolism, cytokine, and chemokine production [13]. Although PLA_2_-generated bioactive lipids, which include FAs, PUFA-derived eicosanoids, and LysoPLs are central to many critical macrophage functions, they have not been extensively studied concerning macrophage activation and functions. Because Pla2g5 is expressed in macrophages and induced by IL-4, it is an attractive target to understand the contribution of bioactive lipids to macrophage polarization and functions. Here we performed a comprehensive analysis of PL, LysoPLs, FAs, and eicosanoids produced by BM-Macs under polarizing conditions, namely LPS+IFNγ and IL-4 (Figure 6).

Analysis of phospholipids in activated BM-Macs showed that PC and PE in BM-Macs are significantly reduced respectively in (LPS+IFNγ)BM-Macs and (IL-4)BM-Macs. Major species including 34:1 PC and 36:2 PE were metabolized in both M1 (LPS+IFNγ) and M2 (IL-4) macrophages, which underscores the importance of lipid metabolism in macrophage activation. LPS+IFNγ was more effective than IL-4 in reducing saturated PC species, including PC 30:0 and PC 32:0. These data are in line with reports in RAW264.7 mouse cell line and human monocyte-macrophages [39,43], although the overall composition of membrane phospholipids in RAW264.7 showed a robust content of PA and PI at baseline, while in human monocyte-derived macrophages PS, PI, and PG contributed to PL composition and changes after activation [44]. Furthermore, IL-4 was more effective on PE metabolites than LPS+IFNγ, while both LPS+IFNγ and IL-4 metabolized PE 38:4 PE and PE 38:5, two molecules that generate AA (20:4) by the action of PLA_2_s.

Macrophage functions are regulated by several PLA_2_s, including Pla2g5, Pla2g4a, Pla2g10, and Pla2g2d which may contribute to PL remodeling during activation [26,43,45]. Notably, Pla2g5 mRNA is induced in macrophages by IL-4 but not LPS+IFNγ [20,32,33]. Indeed, *Pla2g5*-null BM-Macs had a significant increase in several PE molecules in (IL-4)BM-Macs. During LPS+IFNγ activation, *Pla2g5*-null BM-Macs had an increase only in PC 34:1, PC 36:2, and few PE molecules increased during IL-4 activation. Pla2g5 protein is expressed in resting macrophages and translocates following stimulation with pathogens [28]. The effects of Pla2g5 activity in (LPS+IFNγ)BM-Macs are likely due to direct action of preexistent Pla2g5 on membrane PLs or through activation of Pla2g4a, constitutively expressed by macrophages [28] or other PLAs expressed in BM-Macs.

The analysis of LysoPLs did not show increased production of any LysoPLs in activated BM-Macs or a reduction in BM-Macs lacking Pla2g5. In our experiments, IL-4 did not increase the amount of LysoPL molecules in WT BM-Macs. LysoPLs could likely be re-acetylated during activation [46]. Alternatively, Pla2g5 could exert its functions on membrane lipids earlier than 24 h, which is the time point used for macrophage activation [6]. However, it has been reported that macrophages derived from peripheral blood mononuclear cells and activated by IL-4 have reduced LysoPE but not LysoPC when depleted of PLA2G5 by siRNA and activated by IL-4 [33]. Our results also show a preference for Pla2g5 to target PE rather than PC during IL-4 activation of BM-Macs, but we did not detect a reduction of LysoPE molecules in *Pla2g5*-null BM-Macs. Differences in the type of macrophages and depletion of Pla2g5 may account for the discrepancies.

Given our previous data showing that LA and OA generated from macrophages induce pulmonary inflammation [34], we would have expected a decrease at least in selected PUFAs and MUFAs in BM-Macs lacking Pla2g5, particularly in IL-4 activation. Instead, in WT BM-Macs, despite the reduction in PC and PE species containing PUFAs and MUFAs, we could not detect an increase in FAs, making it difficult to detect a decrease in FAs in *Pla2g5*-null BM-Macs. However, FAs can serve as second messengers by binding to cognate receptors, they can be metabolized to supply energy to the cell, re-acylated into the membrane, or serve as substrate to generate eicosanoids [11,12,14,46,47]. Therefore, in cells activated in vitro, FAs are likely heavily used. However, our data showed that PA, the most abundant FA produced in either IL-4 or LPS+IFNγ activation, is reduced in *Pla2g5*-null (LPS+IFNγ)BM-Macs compared to equally treated WT BM-Macs. These results agree with the release of PA by Pla2g5 from other cell types during type 1 inflammation [48,49]. Surprisingly, (IL-4)BM-Macs lacking Pla2g5 had a significant increase in MA (14:0) compared to WT (IL-4)BM-Macs. As MA can be incorporated into a protein with consequences on signal transduction and AA metabolism, future studies await to ascertain the relevance of *Pla2g5*-generated MA in macrophage functions.

The reduction of PE 38:4 and 38:5 in *Pla2g5*-null BM-Macs suggests that, although the analysis of FAs does not support a contribution of Pla2g5 to the generation of AA or other PUFAs, it is likely that eicosanoid generation requires Pla2g5. In macrophages, prostanoids are abundantly produced following TLR-4 stimulation [50]. Furthermore, LPS stimulation increases eicosanoid generation by increasing cPLA_2_α activation and COX-2 expression [51,52,53]. Our data show that PGD_2_ was the most abundant eicosanoid produced by (U)BM-Macs and (LPS+IFNγ)BM-Macs, as reported for RAW264.7 cells [53]. Furthermore, the absence of Pla2g5 significantly reduced PGE_2_ and PGD_2_ in IL-4 and (LPS+IFNγ)BM-Macs, although the effect of LPS+IFNγ was more robust. However, in (LPS+IFNγ)BM-Macs, there was a reduction in 12-HHTrE, an eicosanoid reportedly produced by LPS activated macrophages [54]. These data suggest that the overarching effect of Pla2g5-generated eicosanoids during LPS+IFNγ or IL-4 activation depends, in the former, on the combined action of at least PGD_2_, PGE_2_, and 12-HHTrE, in the latter on PGD_2_, PGE_2_, and 20CooH AA. Indeed, 20CooH AA was significantly induced (IL-4)BM-Macs and reduced in *Pla2g5*-null (IL-4)BM-Macs. 20CooH AA is produced from 20-HETE, a product of CYP ω-oxidation. It is a vasoactive metabolite and activator of PPARγ and PPARα and, therefore, could play a role in Pla2g5-mediated IL-4-induced activation of macrophages [55]. These data indicate that compared with LPS+IFNγ, IL-4 stimulation of BM-Macs results in a weaker COX induction while also activating lipid ω-oxidation, at least partially. The fact that 20CooH AA is increased in *Pla2g5*-null (LPS+IFNγ)BM-Macs underscores the importance of Pla2g5 in the balance between lipid metabolites generated during LPS+IFNγ activation of BM-Macs (COX and 12-15LOX derived) and IL-4 activation (COX and CYP derived). The importance of 20CooH AA in macrophages expressing Pla2g5 is also supported by our data showing that (IL-4)h-Macs lacking PLA2G5 have reduced production of 20CooH AA. In another report, human monocyte-derived macrophages activated with IL-4 and deprived of PLA2G5, did not show any eicosanoid reduction. Our protocol involves monocytes’ isolation by negative selection and culture of the cells in GM-CSF for 10 days to achieve high TGM2 expression [7] and PLA2G5 expression [20]. Therefore, the discrepancies may depend on protocols adopted. However, the relevance of 20CooH AA in any macrophage functions related to Pla2g5 needs to be validated likely by in vivo experiments using mouse models.

Lipoxygenase derived lipids, including Cys-LTs, are potent proinflammatory mediators which are generated by dendritic cells, mast cells, macrophages and Th2 cells. In BM-Macs, we could not detect Cys-LTs (data not shown), likely because the delayed stimulation of 24 h necessary for macrophage polarization in vitro prevents the detection of Cys-LTs. Our data are in line with reports in RAW264.7 cells in which stimulation with Kdo2-Lipid A, a lipopolysaccharide, induces the COX pathway and downregulates the 5-LOX pathway [56]. Furthermore, it cannot be excluded that exogenous Pla2g5 may induce CysLTs generation [57].

The profile of lipids of BM-Macs in one-time point and in *Pla2g5*-null BM-Macs provides a snapshot of the function of a lipid-packed, heterogeneous cell, like macrophages. It does not consider other PLA_2_s expressed in macrophages, including Pla2g4a, Pla2g2d, and Pla2g10, or other sources of exogenous Pla2g5. However, an imbalance between pro and anti-inflammatory lipids could account for the different role of Pla2g5 in the pathogenesis of several pathologies. Additionally, the expression of Pla2g5 in hematopoietic vs. non-hematopoietic cells could also determine the function of Pla2g5 in different diseases [25,26]. A recent study showed that in endothelial cells, the expression of Pla2g5 mRNA is higher than other PLA_2_s (Pla2g1b, 2a, 2d, 2e, 2f, and 10) then its expression is reduced by Angiotensin II stimulation while Pla2g5 protein is still present on the cell surface, likely linked to proteoglycans [49]. On the other hand, in resting macrophages, Pla2g5 mRNA is almost undetectable and is induced by IL-4, while the protein, located intracellularly in resting macrophages, is secreted upon activation [20,29]. As macrophages are hematopoietic derived cells while endothelial cells are derived from mesodermal cells, it is likely that subcellular location and cell ontogeny could also predict Pla2g5 pro or anti-inflammatory functions.

## 5. Conclusions

The combined proinflammatory or anti-inflammatory properties of the lipids produced by M1 (LPS+IFNγ) or M2 (IL-4) activated macrophages may contribute to the functions of macrophages in different diseases. The identification of a potential lipid signature in M1 and M2 macrophages could identify new pathways critical for the development, persistence, or reduction of inflammation. Some of these functions may rely on the presence or absence of Pla2g5 in macrophages.

## Supplementary Materials

The following are available online at https://www.mdpi.com/2218-273X/11/1/25/s1, Figure S1: LysoPL produced by activates BM-Macs.
