# Supplementary material for: Lipid Profile of Activated Macrophages and Contribution of Group V Phospholipase A2
Source: Biomolecules. 2020 Dec 29;11(1):25. doi: 10.3390/biom11010025 (PMC7823364; doi:10.3390/biom11010025)
Supplement: Supplementary file 1 [file biomolecules-11-00025-s001.pdf]

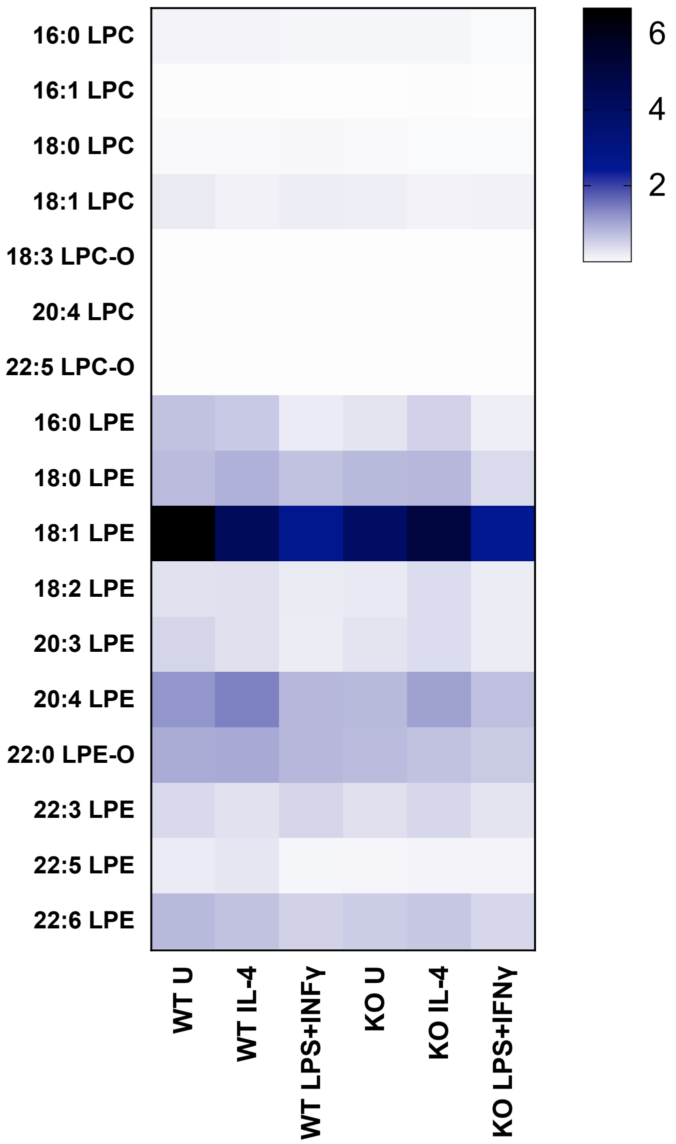

Supplemental Figure 1: Heatmap of LysoPE and LysoPC molecules (as percentages of total PL) produced by WT and Pla2g5-null BM-Macs unstimulated (U) or activated with IL-4 or LPS+IFNγ, analyzed by LC-MS.
